# Using a linked database for epidemiology across the primary and secondary care divide: acute kidney injury

**DOI:** 10.1186/s12911-017-0503-8

**Published:** 2017-07-11

**Authors:** M. Johnson, H. Hounkpatin, S. Fraser, D. Culliford, M. Uniacke, P. Roderick

**Affiliations:** 1 NIHR CLAHRC Wessex Methodological Hub, Faculty of Health Sciences, University of Southampton, Southampton, UK; 2 NIHR CLAHRC Wessex, Primary Care and Population Sciences, Faculty of Medicine, University of Southampton, Southampton, UK; 3 Primary Care and Population Sciences, Faculty of Medicine, University of Southampton, Southampton, UK; 4 Wessex Kidney Centre, Queen Alexandra Hospital, Portsmouth, UK

**Keywords:** Acute kidney injury, Epidemiology, NHS AKI algorithm, Linked data

## Abstract

**Background:**

NHS England has mandated the use in hospital laboratories of an automated early warning algorithm to create a consistent method for the detection of acute kidney injury (AKI). It generates an ‘alert’ based on changes in serum creatinine level to notify attending clinicians of a possible incident case of the condition, and to provide an assessment of its severity. We aimed to explore the feasibility of secondary data analysis to reproduce the algorithm outside of the hospital laboratory, and to describe the epidemiology of AKI across primary and secondary care within a region.

**Methods:**

Using the Hampshire Health Record Analytical database, a patient-anonymised database linking primary care, secondary care and hospital laboratory data, we applied the algorithm to one year (1st January-31st December 2014) of retrospective longitudinal data. We developed database queries to modularise the collection of data from various sectors of the local health system, recreate the functions of the algorithm and undertake data cleaning.

**Results:**

Of a regional population of 642,337 patients, 176,113 (27.4%) had two or more serum creatinine test results available, with testing more common amongst older age groups. We identified 5361 (or 0.8%) with incident AKI indicated by the algorithm, generating a total of 13,845 individual AKI alerts. A cross-sectional assessment of each patient’s first alert found that more than two-thirds of cases originated in the community, of which nearly half did not lead to a hospital admission.

**Conclusion:**

It is possible to reproduce the algorithm using linked primary care, secondary care and hospital laboratory data, although data completeness, data quality and technical issues must be overcome. Linked data is essential to follow the significant proportion of people with AKI who transition from primary to secondary care, and can be used to assess clinical outcomes and the impact of interventions across the health system. This study emphasises that the development of data systems bridging across different sectors of the health and social care system can provide benefits for researchers, clinicians, healthcare providers and commissioners.

**Electronic supplementary material:**

The online version of this article (doi:10.1186/s12911-017-0503-8) contains supplementary material, which is available to authorized users.

## Background

Acute Kidney Injury (AKI) is a rapidly occurring decline in kidney function, associated with poor clinical outcomes and high burden to the health system. Recent years have seen growing interest in the development of methods to facilitate early detection, diagnosis and intervention, enabling clinicians to provide more suitable and timely care for patients with the condition, thereby resulting in improved clinical outcomes. It has been argued that better use of existing clinical information is key to realising this objective [1].

Seeking to create a standard definition of AKI and a consistent method for its detection in a clinical setting, the NHS England automated early warning algorithm (mandated for use by hospital laboratories from March 2015, although implemented sooner in some areas) unifies a number of diagnostic approaches [2, 3]. It is designed to detect possible incident cases of AKI based upon absolute or relative increase above a baseline serum creatinine (SCr) value (drawn from the preceding 48 h, 7 days or 8–365 days), to generate an AKI ‘alert’, and to provide an assessment of severity at the point of testing, as described in Table 1.

**Table 1:** Criteria used to detect incident AKI and allocate an alert stage

AKI Alert Stage	Calculation Criteria
AKI 1 (Low RVR)	Higher RVR < 1.5 and rise in SCr value of >26 μmol/L within preceding 48 h
AKI 1 (High RVR)	Higher RVR ≥ 1.5 and <2
AKI 2	Higher RVR ≥ 2 and <3
AKI 3	Higher RVR ≥ 3 Or higher RVR ≥ 1.5 and SCr ≥ 354 μmol/L

Although AKI research has often focussed on incident cases amongst patients admitted to hospital, the condition can also arise in the outpatient setting [4, 5], or ‘community’ in the UK context. Cases originating in the community may not lead to hospitalisation, but since biochemistry investigations are undertaken by hospital laboratories on behalf of community healthcare providers, the algorithm is able to detect AKI originating in either setting. We hypothesised that reproducing the algorithm using population-level secondary data could enable evaluation of clinical records sourced from both hospital and community settings, thus improving AKI detection and providing better understanding of the epidemiology of the condition, which may in turn better inform clinical practice. We therefore aimed to use the published AKI algorithm as a framework within which to explore the feasibility of using a linked database of routinely collected clinical data to identify the incidence and characteristics of both hospital- and community-acquired AKI (HA-AKI and CA-AKI respectively) across a region.

## Methods

### Study

This study forms part of a broad programme of work in this area, including evaluation of interventions and outcomes both pre- and post-implementation of the algorithm and evaluation of education and working practices; a complete study protocol is publicly available [6]. The study received appropriate ethical approval from the University of Southampton Faculty of Medicine Research Ethics Committee (Submission ID: 15753). Governance approval to access the source data was obtained from the Hampshire Health Record Information Governance Group (HHRIGG).

### Setting

The study was undertaken in the county of Hampshire, southern England; a heterogeneous county with urban population centres around the two major cities on its south coast, and a mixed urban/rural county population. The Hampshire Health Record (HHR) was created with the aim of developing clinical information sharing across the county. It is a live electronic health record allowing clinicians from various care settings to view a consenting patient’s linked medical history, connecting them with data to which they would not otherwise have access and thereby enabling informed decision-making on patient care. HHR links data from participating primary care practices, acute hospitals (including laboratory data from two hospitals) and some data from community and social care providers.

The Hampshire Health Record Analytical database (HHRA) is a separate patient-centric anonymised database created for research and analysis to support health improvement and planning. Although they are separate systems created for different purposes, some content is automatically pushed from HHR to HHRA on a monthly basis and augmented with additional data from other sources. The data are deterministically linked on import to the database using patient NHS number as the unique identifier, and then anonymised before being made available for secondary analysis in a safe-haven environment. Additional file 1: Figure 1 shows the source data used for this study, and the means of collection via HHR and HHRA.

At the time of the present study, HHRA included data for 146 practices and around 1.4 million patients, representing coverage of approximately 75% of the resident Hampshire population. Although not all local practices participate, those that are missing are dispersed across the catchment area, with varied rural/urban classification, socioeconomic deprivation and patient composition. We are not aware of any systematic differences to those practices whose data are present. The linked data sources within HHRA include primary care (coded clinical entries made during routine patient care), secondary care (inpatient, outpatient and emergency department activity data sourced from the Secondary Uses Service [7]) and hospital laboratories.

Both HHR and HHRA are hosted and maintained by NHS South, Central and West Commissioning Support Unit. The governance body is the HHRIGG, which ensures the security and confidentiality of both systems and considers issues of data integration and sharing.

### Data sources

All hospital laboratories in England use a Laboratory Information Management System (LIMS) for administration and recording of test results. To standardise the implementation and operation of the algorithm across the country, LIMS providers have created applications to directly incorporate it into their systems [2, 8]. This allows the algorithm to run natively and automatically within LIMS at the point of SCr testing, evaluating each result individually to detect incident AKI and, if present, generate an alert and indicate its severity.

Laboratory test dates and results (including SCr) recorded by two local hospitals are pushed from LIMS to HHRA, via HHR. This, linked to data sourced from primary and secondary care settings, provides all of the information needed to replicate the algorithm wholly within the HHRA environment, without the need for ad hoc data collection from hospital laboratories or healthcare providers. The HHRA database has a relational structure, and query code has been written using Microsoft Structured Query Language (SQL) Server 2008 R2 to assemble the requisite information and run the algorithm.

In the UK Read Codes are the most common method of coding clinical activity taking place in primary care, and are sometimes also used in other care settings [9]. There are two versions currently in use: Read Codes Version 2 (Read v2) and Clinical Terms Version 3 (CTV3), of which Read v2 is most widely used in Hampshire. One of the hospital laboratories contributing to HHRA records tests using Read v2 whilst the other uses a local coding system. To facilitate interoperability, consistency across sectors and efficient replication of database query routines, local codes are mapped to Read v2 on import into HHRA.

### Cohort selection

Since the algorithm is predicated on the use of multiple longitudinal SCr values to establish a baseline, complete historical data are essential. Although HHRA contains SCr results covering our entire study period (a combined observation year of 1st January-31st December 2014; and ‘look-back’ period of 365 days for each SCr, 1st January-31st December 2013), coverage was not complete across the entire county, necessitating development of a local solution.

While participating practices are distributed across Hampshire, only those hospital laboratories in the cities of Southampton and Portsmouth contribute data. The majority of tests are requested either internally by an attending clinician whilst the patient is in hospital or at distance by general practice. Since laboratories are more likely to undertake biochemical investigations for patients resident within a relatively close proximity, contractual and geographical factors determine which practices are regular users of those located in Southampton or Portsmouth. This, in turn, influences the likelihood of individual patients having a complete, longitudinal linked record available in HHRA for the entire duration of the study period.

We identified biochemical tests associated with practices located in Southampton and Portsmouth and calculated testing rates for each to establish their typical profile of laboratory usage. Following the method of identifying and excluding outliers proposed by Tukey [10], the lower fence of the distribution (calculated as the lower quartile less one and a half times the interquartile range) was set as an inclusion threshold. Having then calculated biochemical testing rates for all other participating practices in Hampshire, we excluded any (including those in Southampton and Portsmouth) whose rate fell below this threshold. This reduced the initial number of 146 participating practices to a subset of 94 practices empirically found to be regular users, each of which was located within a reasonable proximity of Southampton or Portsmouth hospital laboratories.

Thereafter, individual patients were selected from those practices based on additional eligibility criteria. We included all patients aged 18 or over as at the start of the observation year and who were registered within the subset of viable practices throughout the entire study period. This amounted to a total of 642,337 patients included in the final study cohort. Additional file 2: Figure 2 summarises the practice and patient cohort selection process.

### Query procedures

The algorithm iteratively compares individual SCr results against two distinct reference values, each dynamically selected from a defined time period:

i) The lowest SCr value within 0–7 days preceding the present result; or
ii) The median SCr value within 8–365 days preceding the present result.

In each case, a reference value ratio (RVR) is calculated as the present SCr value divided by the reference value. The present SCr value is then compared to the higher RVR to detect AKI and allocate an alert stage, as shown in Table 1. An AKI alert is generated where the RVR is greater than or equal to 1.5, but can also be generated where it is less than 1.5 and there has been an SCr rise of more than 26 μmol/L within the 48 h period preceding the present test result.

We have written a series of SQL query procedures to iteratively assess each SCr recorded during the observation year for our entire cohort of 642,337 patients, and allocate an alert stage where appropriate. We have also written procedures to link laboratory data to antecedent primary care and subsequent secondary care data, enabling evaluation of AKI determinants, incidence and outcomes, and classification of each incident AKI episode as HA-AKI or CA-AKI based upon inpatient hospitalisation dates.

## Results

Of the 642,337 patients in our study cohort 176,113 (27.4%) had at least two SCr values available (the minimum number required to detect incident AKI). We identified 5361 patients (approximately 0.8%) generating at least one AKI alert of any stage during the observation year, to an overall total of 13,845 alerts. An age gradient was present in the pattern of SCr tests; the majority of patients aged 65 or over had two or more SCr values available, and while most of the patients with multiple SCr values were in these age groups, most of those with one or none were younger. The corollary to this was a similar gradient in AKI alerts, with a higher likelihood amongst older age groups. A full breakdown of SCr tests and AKI alerts by gender and age is shown in Table 2.

**Table 2:** Number and demographic characteristics of SCr tests and AKI alerts

	All patients	Percentage of the cohort with specified number of SCr values available		Patients with 1+ AKI alert (any stage) during observation year		Number of AKI alerts (any stage) during observation year
		0–1	2+	Number	% of cohort	
Cohort population	642,337	72.6	27.4	5361	0.8	13,845
Gender						
Male	312,896	74.8	25.2	2359	0.8	6860
Female	329,441	70.5	29.5	3002	0.9	6985
Age						
18–24	67,147	92.5	7.5	99	0.1	193
25–34	100,268	90.2	9.8	216	0.2	418
35–44	102,900	86.3	13.7	230	0.2	510
45–54	120,008	78.6	21.4	401	0.3	1177
55–64	98,253	66.5	33.5	656	0.7	1877
65–74	83,460	49.4	50.6	1088	1.3	3154
75–84	49,808	34.8	65.2	1501	3.0	3693
85–94	19,087	31.8	68.2	1083	5.7	2645
95+	1406	44.2	55.8	87	6.2	178

All percentages relate to rows and have been rounded to one decimal place

We stratified CA-AKI alerts into three types: those originating from an SCr taken in the community and not leading to a hospital admission, those from an SCr taken in the community where an admission occurred within the next seven days, and those alerted on admission to hospital (having therefore originated beforehand). Taking each patient’s first alert during the observation year as a cross-section of the distribution of alert types, we found that more than two-thirds originated in the community, of which nearly half did not lead to an admission, and nearly half were triggered on inpatient admission. By contrast, when all alerts during the observation year were considered, we found that a higher proportion were generated in hospital. A full breakdown of AKI alerts by type is shown in Table 3.

**Table 3:** AKI type of alerts (any stage) occurring during the observation year

AKI type	First alert		All alerts	
	Number	% of total	Number	% of total
Not admitted CA-AKI	1652	30.8	2892	20.9
CA-AKI admitted within 7 days	405	7.6	653	4.7
CA-AKI identified on admission	1575	29.4	1876	13.6
All CA-AKI	3632	67.8	5421	39.2
All HA-AKI	1729	32.3	8424	60.8
Total	5361	100.1^a^	13,845	100.0

All percentages relate to column totals and have been rounded to one decimal place

^a^Total does not equal 100.0 owing to rounding

## Discussion

### Identifying AKI in hospital and community settings

We have successfully applied the NHS England AKI early warning algorithm retrospectively across a large, regional population. A recent development, research utilising the algorithm in a secondary data environment is currently sparse, and we believe that the present study is one of the first attempts to reproduce it at a population level using retrospective data. In so doing, we have also demonstrated the feasibility and importance of using linked data to describe the epidemiology of AKI. Although it is possible to use primary or secondary care data individually to identify AKI that is acquired and remains in the community or in hospital alone, we have shown that linked data is essential to follow the significant proportion of people with AKI who frequently cross the primary/secondary care divide.

Importantly, we found that more than two-thirds of first alerts related to AKI originating in the community, of which nearly half did not lead to admission, therefore describing a significant proportion of incident cases that may not be detectable or fully understood without data sourced from the community setting. Beyond more effective detection of AKI, linked hospital and community data has also given us the ability to better describe the pathway to detection than would be possible if focussing only upon hospital admission data. Similarly, implementation across a region has given us an integrated perspective that could not be achieved in the clinical environment of a single laboratory. In this scenario only those biomarkers generated and recorded by the hospital itself can be evaluated by the algorithm, potentially limiting the accuracy of its results if relevant information is omitted. The use of linked data in this way could therefore represent an important regional complement to the in-hospital, clinical use of the AKI algorithm.

### Establishing a complete record

Despite the limitation of incomplete coverage of the county of Hampshire, a key strength of this study was its use of a linked database embedded within the local health system. Access to local, linked data has facilitated and added value to our work, which, alongside previous research in a variety of clinical areas using the same data source [11–14], further emphasises the clinical and research benefits of data sharing within and amongst contiguous geographical localities.

Having used data sourced directly from NHS information systems, we have assumed that all data are complete. Validation of this assumption was not possible as the governance in this area is difficult, particularly for researchers. Nevertheless, a strength of this study was the availability of a substantial number of usable SCr records for our population of interest. Previous studies retrospectively detecting AKI incidence have dealt with missing SCr values using a variety of methods [15–20], but our data source has overcome this limitation, eliminating the need to find alternative means to establish an SCr baseline. Despite this, we acknowledge that systematic issues related to the collection of SCr values have the potential to distort our results. Both a baseline and comparative SCr value are required to generate an alert, and if not available it would be impossible for the algorithm to detect AKI, even if clinically present. The potential for a proportion of patients to remain undetected on the basis of insufficient data, resulting in population-level underestimation of AKI, therefore persists if technical issues have prevented SCr values’ appearance in the source data, or if blood samples were simply never collected. This may be disproportionately true of certain demographics, including males, and those in younger or healthier groups. The potential significance of each of these factors can be discerned from our results, which indicate an age gradient in the frequency of SCr testing, and show that the minority of patients had both a baseline and comparative SCr value available (the minimum required to detect incident AKI). At the same time, the higher frequency of biochemical investigations amongst hospitalised patients as compared to those in the community, creates a higher likelihood of AKI detection amongst this population and parallel under-estimation of that acquired in the community [5, 21, 22]. Moreover, given its principal purpose of indicating incident AKI in a clinical environment, the algorithm is designed to generate repeated alerts throughout the duration of an AKI episode. We also acknowledge, therefore, that use for research demands further clinical interpretation of the output to isolate distinct episodes.

Another limitation to this study, as seen elsewhere [21, 23], is that missing time stamps on a significant proportion of SCr values has necessitated our deviation from the published algorithm. This precludes assessment of the precise order of events, and limits the ability to consistently and reliably observe the time of the test. The ‘AKI 1 (Low RV)’ criteria require detection of a rise of 26 μmol/L within the preceding 48 h period, but, to accommodate this limitation, we have instead used a two day period as a proxy, with potential to overestimate incident AKI.

Data quality remains an issue when working with large-scale linked databases, which are often prone to variation and inconsistencies associated with the aggregation of data created by multiple users and sourced from different systems and sectors [24–26]. ‘Real-world’ patient records, in many cases populated by clinical users for the primary purpose of direct patient care, may also vary longitudinally in response to system implementation and changing patterns of clinical behaviour [27, 28]. That HHRA collects data produced by many local healthcare providers, each individually responsible for its own data collection, therefore inherently drives variation [26, 29]. We have written a series of SQL procedures to modularise the processes of assembling information and replicating the algorithm, implementing checks for many of the common data quality issues [30] throughout to identify errors and either rectify or isolate them where appropriate. Our SQL procedures are relatively simple, and can be reproduced in any system linking hospital laboratory to primary and secondary care data. Challenges arose in staging and sequencing the procedures to accommodate the volume of data, as the need to retrieve and order historical SCr values for all members of a substantial patient cohort imposed significant demand on the HHRA working environment. As well as facilitating error trapping of data quality issues, a modular approach to writing SQL procedures also helped in this regard. We believe that further improvements could be achieved if a dedicated server and current database management software were used.

### Clinical implications

A recent study [21] to validate algorithm-detected AKI against that already diagnosed by a nephrologist demonstrated that the algorithm performs well, and we have shown that it can also be applied retrospectively to detect incident AKI in the general population. Our results confirm previous findings that AKI may arise more commonly in the community than in hospital [5, 17, 22], again underlining the importance of an approach that considers both settings.

The scope of this component of our broader work programme was to demonstrate the feasibility of such an approach, but our work also highlights the potential and need for further research to better understand the epidemiology of AKI. For example, availability of data from both hospital and community settings could enable comparison of the severity of the condition, resource use and costs across the health system, and patient outcomes amongst those with HA-AKI and those with (admitted or non-admitted) CA-AKI. Furthermore, although Read v2 codes to record primary care diagnosis of AKI were not in use at the time of our secondary analysis, their subsequent implementation, and future availability, will enable consideration of how alerting in hospital or primary care translates into changes in patient management across clinical settings. Similarly, as the present study relates to a time prior to widespread implementation of AKI alerting in hospital, further research could compare post-implementation data to evaluate alerting as an intervention. Finally, our approach could be further developed by way of linkage to additional data sources hitherto unavailable to our study group, such as in-hospital system data concerning clinical observations, which help to reveal the determinants of related clinical events.

We also recognise that each individual alert cannot be assumed to represent a distinct AKI episode. In fact, that our results indicate a high proportion of HA-AKI alerts may reflect the propensity for repeated biochemical investigations during an inpatient spell to generate multiple, serialised alerts related to a single AKI episode. It may be, therefore, that alerts are not a true epidemiological measure of AKI, and work is ongoing in our group to investigate patterns of AKI alerts and episodes, recovery and recurrence.

## Conclusions

We have demonstrated the feasibility of using linked data from hospital and community settings to reproduce the NHS England automated early warning algorithm, having identified the incidence and characteristics of both HA-AKI and CA-AKI in a regional population. Considerable effort was required to overcome technical and data quality issues, but having worked as a multidisciplinary team drawing upon informatics and clinical expertise was an advantage, allowing us to investigate and make clinical judgments based upon ‘real-world’ data rather than relying upon application of standard clinical rules.

This study underlines the potential benefits of using linked data to research the epidemiology of a condition that frequently crosses the primary/secondary care divide, but also highlights issues around data sharing, data quality and system interoperability, and the wider benefits of developing healthcare data systems that can bridge across different sectors of the health and social care system. In AKI, linked data could represent an important regional complement to the clinical use of the algorithm, but could also be instrumental in the development and validation of tools to predict risk in the general population, thus demonstrating the value that informatics can bring to the overall aims of high quality clinical management of AKI and its prevention in those at high risk of the condition.

## Additional files

Additional file 1: Figure 1. Conceptual diagram of HHR, HHRA and the source data used for this study. (DOCX 67 kb)

Additional file 2: Figure 2. Summary of patient and practice selection process. (DOCX 63 kb)

## Abbreviations

AKI: Acute kidney injury
CA-AKI: Community-acquired acute kidney injury
CTV3: Clinical Terms Version 3
HA-AKI: Hospital-acquired acute kidney injury
HHR: Hampshire Health Record;
HHRA: Hampshire Health Record Analytical database
LIMS: Laboratory Information Management System
NHS: National Health Service
NIHR: National Institute for Health Research
Read v2: Read Codes Version 2
RVR: Reference value ratio
SCr: Serum creatinine
SQL: Structured Query Language

## References

[1] Thomas M, Sitch A, Dowswell G (2011). The initial development and assessment of an automatic alert warning of acute kidney injury. Nephrol Dial Transplant.

[2] NHS England: Acute Kidney Injury (AKI) Algorithm. https://www.england.nhs.uk/patientsafety/akiprogramme/aki-algorithm/ (2016). Accessed 25 Oct 2016.

[3] Think Kidneys: Acute Kidney Injury Warning Algorithm Best Practice Guidance. https://www.thinkkidneys.nhs.uk/wp-content/uploads/2014/12/AKI-Warning-Algorithm-Best-Practice-Guidance-final-publication-0112141.pdf (2014). Accessed 25 Oct 2016.

[4] Feest TG, Round A, Hamad S (1993). Incidence of severe acute renal failure in adults: results of a community based study. BMJ.

[5] Wonnacott A, Meran S, Amphlett B, Talabani B, Phillips A (2014). Epidemiology and outcomes in community-acquired versus hospital-acquired AKI. Clin J Am Soc Nephrol.

[6] NIHR CLAHRC Wessex: the Hampshire acute kidney injury study. http://www.clahrc-wessex.nihr.ac.uk/img/projects/CLAHRC_AKI_protocol_FINAL.pdf (2016). Accessed 30 June 2016.

[7] NHS Digital: Secondary uses service (SUS). http://content.digital.nhs.uk/sus (2016). Accessed 16 Feb 2017.

[8] Thomas ME, Blaine C, Dawnay A, Devonald MA, Ftouh S, Laing C, Latchem S, Lewington A, Milford DV, Ostermann M (2015). The definition of acute kidney injury and its use in practice. Kidney Int.

[9] Benson T (2011). The history of the read codes: the inaugural James read memorial lecture 2011. J Innov Health Informatics.

[10] Tukey J (1977). Exploratory data analysis.

[11] Fraser SD, Parkes J, Culliford D, Santer M, Roderick PJ (2015). Timeliness in chronic kidney disease and albuminuria identification: a retrospective cohort study. BMC Fam Pract.

[12] Fraser SDS, Watkinson GE, Rennie CA, King D, Sanderson H, Edwards L, Roderick P. Sociodemographic differences in diabetic retinopathy screening; using patient-level primary care data for health equity audit. Clin Audit. 2011;7

[13] Sundvall P, Stuart B, Davis M, Roderick P, Moore M (2015). Antibiotic use in the care home setting: a retrospective cohort study analysing routine data. BMC Geriatr.

[14] Williams NP, Coombs NA, Johnson MJ, Josephs LK, Rigge LA, Staples KJ, Thomas M, Wilkinson TM (2017). Seasonality, risk factors and burden of community-acquired pneumonia in COPD patients: a population database study using linked health care records. Int J Chron Obstruct Pulmon Dis.

[15] Kerr M, Bedford M, Matthews B, O'Donoghue D (2014). The economic impact of acute kidney injury in England. Nephrol Dial Transplant.

[16] Lafrance JP, Djurdjev O, Levin A (2010). Incidence and outcomes of acute kidney injury in a referred chronic kidney disease cohort. Nephrol Dial Transplant.

[17] Selby NM, Crowley L, Fluck RJ, McIntyre CW, Monaghan J, Lawson N, Kolhe NV (2012). Use of electronic results reporting to diagnose and monitor AKI in hospitalized patients. Clin J Am Soc Nephrol.

[18] Siew ED, Matheny ME, Ikizler TA, Lewis JB, Miller RA, Waitman LR, Go AS, Parikh CR, Peterson JF (2010). Commonly used surrogates for baseline renal function affect the classification and prognosis of acute kidney injury. Kidney Int.

[19] Siew ED, Peterson JF, Eden SK, Moons KG, Ikizler TA, Matheny ME (2013). Use of multiple imputation method to improve estimation of missing baseline serum creatinine in acute kidney injury research. Clin J Am Soc Nephrol.

[20] Závada J, Hoste E, Cartin-Ceba R, Calzavacca P, Gajic O, Clermont G, Bellomo R, Kellum JA (2010). A comparison of three methods to estimate baseline creatinine for RIFLE classification. Nephrol Dial Transplant.

[21] Sawhney S, Marks A, Ali T, Clark L, Fluck N, Prescott GJ, Simpson WG, Black C (2015). Maximising acute kidney injury alerts--a cross-sectional comparison with the clinical diagnosis. PLoS One.

[22] Sawhney S, Fluck N, Fraser SD, Marks A, Prescott GJ, Roderick PJ, Black C (2016). KDIGO-based acute kidney injury criteria operate differently in hospitals and the community-findings from a large population cohort. Nephrol Dial Transplant.

[23] Sawhney S, Fluck N, Marks A, Prescott G, Simpson W, Tomlinson L, Black C (2015). Acute kidney injury-how does automated detection perform?. Nephrol Dial Transplant.

[24] Dobbins TA, Badgery-Parker T, Currow DC, Young JM (2015). Assessing measures of comorbidity and functional status for risk adjustment to compare hospital performance for colorectal cancer surgery: a retrospective data-linkage study. BMC Med Inform Decis Mak.

[25] Michalakidis G, Kumarapeli P, Ring A, van Vlymen J, Krause P, de Lusignan S (2010). A system for solution-orientated reporting of errors associated with the extraction of routinely collected clinical data for research and quality improvement. Stud Health Technol Inform.

[26] van Vlymen J, de Lusignan S, Hague N, Chan T, Dzregah B (2005). Ensuring the quality of aggregated general practice data: lessons from the primary care data quality Programme (PCDQ). Stud Health Technol Inform.

[27] John A, McGregor J, Fone D, Dunstan F, Cornish R, Lyons RA, Lloyd KR (2016). Case-finding for common mental disorders of anxiety and depression in primary care: an external validation of routinely collected data. BMC Med Inform Decis Mak.

[28] Millett ER, Quint JK, De Stavola BL, Smeeth L, Thomas SL (2016). Improved incidence estimates from linked vs. stand-alone electronic health records. J Clin Epidemiol.

[29] de Lusignan S, van Weel C (2006). The use of routinely collected computer data for research in primary care: opportunities and challenges. Fam Pract.

[30] Berndt DJ, Fisher JW, Hevner AR, Studnicki J (2001). Healthcare data warehousing and quality assurance. IEEE Computer.

