# Supplementary material for: Using a linked database for epidemiology across the primary and secondary care divide: acute kidney injury
Source: BMC Med Inform Decis Mak. 2017 Jul 11;17:106. doi: 10.1186/s12911-017-0503-8 (PMC5504785; doi:10.1186/s12911-017-0503-8)
Supplement: Supplementary file 1 [file 12911_2017_503_MOESM1_ESM.docx]

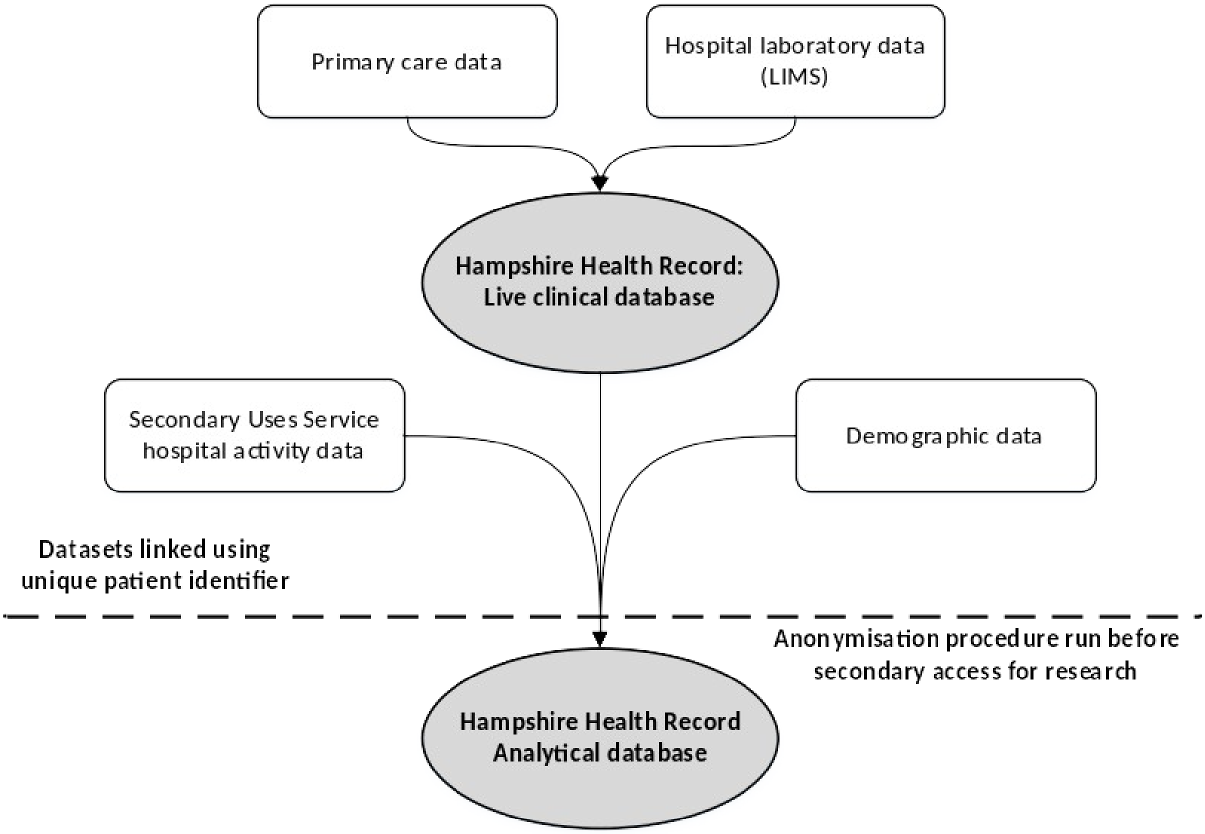
Primary care data
Hospital laboratory data (LIMS)
Hampshire Health Record: Live clinical database
Secondary Uses Service hospital activity data
Demographic data
Datasets linked using unique patient identifier
Anonymisation procedure run before secondary access for research
Hampshire Health Record Analytical database
