# Supplementary material for: Using a linked database for epidemiology across the primary and secondary care divide: acute kidney injury
Source: BMC Med Inform Decis Mak. 2017 Jul 11;17:106. doi: 10.1186/s12911-017-0503-8 (PMC5504785; doi:10.1186/s12911-017-0503-8)
Supplement: Supplementary file 2 [file 12911_2017_503_MOESM2_ESM.docx]

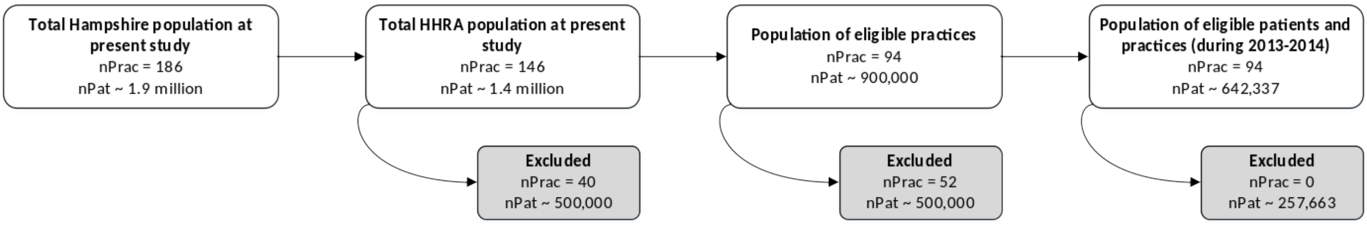
Total Hampshire population at present study
nPrac = 186
nPat ~ 1.9 million
Total HHRA population at present study
nPrac = 146
nPat ~ 1.4 million
Excluded
nPrac = 40
nPat ~ 500,000
Population of eligible practices
nPrac = 94
nPat ~ 900,000
Excluded
nPrac = 52
nPat ~ 500,000
Population of eligible patients and practices (during 2013-2014)
nPrac = 94
nPat ~ 642,337
Excluded
nPrac = 0
nPat ~ 257,663
